# PhenoMIP: High-Throughput Phenotyping of Diverse *Caenorhabditis elegans* Populations via Molecular Inversion Probes

**DOI:** 10.1534/g3.120.401656

**Published:** 2020-08-31

**Authors:** Calvin Mok, Gabriella Belmarez, Mark L. Edgley, Donald G. Moerman, Robert H. Waterston

**Affiliations:** * Department of Genome Sciences, University of Washington, Seattle, 98195; † Department of Zoology, University of British Columbia, Vancouver, Canada, V6T 1Z4

**Keywords:** *Caenorhabditis elegans*, Molecular Inversion Probes, Quantitative Fitness, Million Mutation Project, Multiplex Population, Competitive Fitness Assay

## Abstract

Whether generated within a lab setting or isolated from the wild, variant alleles continue to be an important resource for decoding gene function in model organisms such as *Caenorhabditis elegans*. With advances in massively parallel sequencing, multiple whole-genome sequenced (WGS) strain collections are now available to the research community. The Million Mutation Project (MMP) for instance, analyzed 2007 N2-derived, mutagenized strains. Individually, each strain averages ∼400 single nucleotide variants amounting to ∼80 protein-coding variants. The effects of these variants, however, remain largely uncharacterized and querying the breadth of these strains for phenotypic changes requires a method amenable to rapid and sensitive high-throughput analysis. Here we present a pooled competitive fitness approach to quantitatively phenotype subpopulations of sequenced collections via molecular inversion probes (PhenoMIP). We phenotyped the relative fitness of 217 mutant strains on multiple food sources and classified these into five categories. We also demonstrate on a subset of these strains, that their fitness defects can be genetically mapped. Overall, our results suggest that approximately 80% of MMP mutant strains may have a decreased fitness relative to the lab reference, N2. The costs of generating this form of analysis through WGS methods would be prohibitive while PhenoMIP analysis in this manner is accomplished at less than one-tenth of projected WGS costs. We propose methods for applying PhenoMIP to a broad range of population selection experiments in a cost-efficient manner that would be useful to the community at large.

The *C. elegans* haploid genome is compact, containing just over 100 Mb, and yet is capable of generating a complex organism with a defined cell lineage (Sulston *et al.* 1983). Despite our detailed knowledge of this organism, much of its biology remains unclear. At current, only 9,645 Wormbase genes (Wormbase web site 2019) have phenotype descriptions reported from either variant alleles or RNAi knockdown experiments, suggesting that the function of nearly half of *C. elegans* protein coding genes remain experimentally uncharacterized. Knowledge of where and when a gene is expressed can provide clues to function and many large data sets have elucidated gene expression patterns across embryonic, larval and adult timepoints. Furthermore, multiple techniques have begun to resolve tissue-specific and even cell-specific expression profiles (Boeck *et al.* 2016; Cao *et al.* 2017; Gracida and Calarco 2017; Kaletsky *et al.* 2018; Warner *et al.* 2019). However, this information does not directly reveal gene function *per se*.

Forward genetics screens by methods such as chemical mutagenesis, provide a means of recovering alleles that result in a detectable phenotype of interest such as sterility, lethality, or altered reporter expression. These alleles can then be genetically mapped, sequenced, and functionally analyzed. In this manner, a specific phenotype can be screened across hundreds of thousands of mutated genomes, thereby querying a very large search space (Brenner 1974; De Stasio and Dorman 2001; Kevin *et al.* 2006). The identification of causal variants across this space can be a laborious process although a variety of methods now exist to aid in the sequencing and mapping of mutant genomes (Doitsidou *et al.* 2010; Minevich *et al.* 2012; Jaramillo-Lambert *et al.* 2015; Mok *et al.* 2017). In contrast, a reverse genetics screen by RNAi, generates a smaller potential search space by querying a collection of specific gene knock-down targets for a detectable phenotype in a limited number of genetic backgrounds (Fraser *et al.* 2000; Kamath *et al.* 2003; Lehner *et al.* 2006). Consequently, the solution space is relatively well-defined since validated hits require no genetic mapping, although such screens are generally confined to knocking down gene expression rather than necessarily exploring states of altered protein function. Depending upon assay format, an RNAi screen’s throughput can be comparatively less than a mutagenesis screen. Furthermore its effects may be problematic, producing false negatives or weak hits due to incomplete knockdown or false positives from the knockdown of gene families (Fraser *et al.* 2000; Parrish *et al.* 2000; De-Souza *et al.* 2019). In both screening methods, the ability to score a detectable phenotype may also be affected by the presence of redundant paralogs or entire parallel systems that can compensate for a reduced function (for review see (Jorgensen and Mango 2002)).

Whether as a result of paralogs or other reasons, phenotypes that moderately, weakly or partially affect development or fecundity might be overlooked as stochastic variation while actually influencing overall population fitness (Schnabel *et al.* 1997; Richards *et al.* 2013; Diaz and Viney 2014; Perez *et al.* 2017). Subtle population-wide shifts in phenotypic fitness require quantitative methods of analysis that go beyond low-resolution phenotype qualifiers such as slow-growth, sterile, or lethal. With collections of sequenced strains such as the mutant strains of the Million Mutation Project (MMP) and the wild isolates of the *Caenorhabditis elegans* Natural Diversity Resource (CeNDR) there are now many sequenced alleles across the *C. elegans* genome that could prove informative to deciphering the roles in organismal fitness of uncharacterized and characterized genes alike (Thompson *et al.* 2013; Cook *et al.* 2017). It remains, however, a matter of devising an efficient bulk interrogation method for strains that may only reveal fitness phenotypes under altered conditions such as temperature and food source or through sensitized genetic backgrounds via gene knock down (Hirsh and Vanderslice 1976; Lehner *et al.* 2006; Diaz and Viney 2014). In recent years, strides have been made in the quantitative analysis of fitness (Elvin *et al.* 2011; Ramani *et al.* 2012; Crombie *et al.* 2018). Advances in next generation sequencing technologies have led to a number of quantitative approaches to population analysis of singular genetic backgrounds by comparing deeply-sequenced samples for changes to transcription, small RNA populations, and heterochromatin (Warf *et al.* 2012; Araya *et al.* 2014; Boeck *et al.* 2016; Daugherty *et al.* 2017). More recently, Webster *et al.*, have reported on the restriction site-associated DNA sequencing of pooled *C. elegans* wild isolate populations to identify long-term starvation-selected phenotypes (Webster *et al.* 2019). Alternatively, by tagging mutated populations with specific molecular identifiers, only a portion of the genome requires sequencing, thus reducing the required sequencing burden of an experiment (Smith *et al.* 2009; Levy *et al.* 2015). This form of population barcoding, while relatively new, has brought insights to how initially homogenous populations of yeast competitively evolve over time (Levy *et al.* 2015; Blundell *et al.* 2019). Leveraging current sequencing and population barcoding paradigms to analyze nematode population fitness would improve the process of strain analysis in large collections such as the MMP and CeNDR. In turn, unlocking the effects of the variants harbored within these collections could fast-track the process of assigning function to poorly characterized genes or alleles across this invertebrate genome.

To further expand our knowledge of *C. elegans* gene function, we sought to develop an assay that could 1) mimic the allelic diversity of a forward genetics screen but with a smaller solution space much like a reverse genetics screen and 2) generate quantitative data regarding population fitness to assess potential gene function. We exploited the self-fertilizing hermaphroditic nature of *C. elegans* to grow multiple strains in pools with little or no genetic mixing. We hypothesized that distinct mutations in each strain could be treated as a barcode to identify and quantify the representation of each strain within the pool. To assay the mutations and thus the representation of each strain in these pools, rather than use cost-prohibitive whole genome sequencing, we adapted molecular inversion probes (MIPs) to identify strain-specific variants (Hiatt *et al.* 2013). We previously used MIPs for the genetic mapping of temperature-sensitive alleles in a collection of *C. elegans* mutant strains (Mok *et al.* 2017); here we analyze population growth in a multi-generational competitive fitness assay to phenotype via MIPs (PhenoMIP) by quantifying the proportion of each strain in a pool. As a proof of principle, we utilized the Million Mutation Project as a source for our strains. The MMP library of 2007 N2-derived mutant strains harbors a variety of coding alleles including potential null alleles across 8150 protein-coding genes, and coding or splice site-altering SNVs across 19,666 genes (Thompson *et al.* 2013). The phenotypic consequences for many of these variants remain relatively unexplored; we hypothesized that some may play a role in overall fitness. For each MMP strain, we identified unique genetic markers suitable for detection by MIPs. Using these strain-specific MIPs, we effectively generated barcodes for composition analysis of genotypes within a genetically heterogenous population – analogous to methods used in yeast (Hardenbol *et al.* 2003). We analyzed population composition at multiple timepoints to estimate the relative fitness for each individual strain within a pool, and thereby catalog the potential fitness range of this collection. Our observations suggest that PhenoMIP has the sensitivity to categorize strains into a range of population fitness phenotypes. Overall, we show that PhenoMIP is a cost-efficient and high-throughput approach to the quantitative analysis of pooled mutagenized genomes assayed across multiple experimental conditions.

## Materials And Methods

### MIP site selection and design

MIP sites were selected in two rounds. Initially the entire MMP SNV data set was used to select for sites that were spaced a minimum of 300 bp apart to avoid potential collisions with neighboring probes. Site selection and rejection was completed in a linear manner based on the first available SNV on each linkage group within the data set. Locations were not filtered or optimized to reduce the occurrence of neighboring SNVs within the 300 bp window. The initial set of MMP mutant strain MIP sites was then used to remove candidate sites from the MMP wild isolate data set. Any wild isolate sites within a 350 bp window of mutant candidate sites was removed from selection. Of the remaining wild isolate SNV sites, a 350 bp selection window was used to identify potential MIP sites. The list of candidate MIP sites was used to design and score MIPs based on previously published criteria (Mok *et al.* 2017). The list of designed MIPs was subdivided into each individual strain where the highest-scoring MIP for each linkage group was identified. Of the six MIPs designed for each strain, four were randomly selected for use in population analysis (Supplemental Data SD1).

### MIP library pooling, preparation, and sequencing

MIPs were pooled based on worm pools being tested and generated as previously published (Mok *et al.*, 2017). Individual MIPs were normalized to a concentration of 100 uM and pooled to a maximum volume of 85 ul. 10 ul of 10X Polynucleotide Kinase (PNK) Buffer and 5 ul of PNK were added to a volume of 85 ul pooled MIPs before incubating for 45 min at 37° and 20 min at 80°. This pool was then diluted to a working concentration of 330 nM. MIP libraries were generated with 500 ng genomic DNA template (4.86x10^−6^ genomes) and appropriate MIP pools as previously described in Mok *et al.* 2017. Libraries were sequenced on Illumina MiSeq or NextSeq systems. Libraries across pools ranged between 3.3x10^6^ and 32.7x10^6^ combined reads with an average 1566 reads per probe (Supplemental Data SD5).

### Worm maintenance and pooling

Worms were maintained at 20° on standard nematode growth media (NGM) seeded with OP50. Worm pools were generated from well-fed source plates using exclusively twenty L1 or L4 animals for each strain. Starting pools were grown on 15cm NGM made with 8X peptone and seeded with NA22 or HT115 (transformed with L4440 empty vector) *E. coli* strains. Pools were grown at 20° for 96-120 hr which allowed for a brief period of starvation of less than 24 hr to synchronize any remaining eggs to the L1 stage. Worms were then washed off with 10-15 ml M9, pelleted and aspirated to 5-6 ml before population density was assessed. 50-100 ul of pellet was frozen as a representative sample of the initial pooled population. Pools were then redistributed in equal-sized populations between 5000 and 10000 animals on 15 cm 8X peptone NGM plates that were seeded with bacteria based on the reported experimental conditions. RNAi pooling experiments were carried out on 8X peptone NGM plates supplemented with IPTG to 4mM and carbenicillin at 25 µg/ml. RNAi plates were seeded with ∼500 ul of saturated overnight cultures grown with carbenicillin at 25 µg/ml. For each batch of plates generated, *dpy-11* RNAi control plates were used to ascertain the effectiveness of the RNAi feeding. Populations were grown for ∼96 hr before being transferred to replicate condition plates either by chunking or washing again. At this point, the transferred populations were in a “starvation” state for less than 24 hr, and most remaining eggs hatched and synchronized to approximately the L1 stage. Any remaining animals were washed from plates with double-distilled water, pelleted, and frozen as samples for later analysis. Each cycle of transfer approximately followed a single generation and pooling experiments were propagated for 6-10 generations. Heavily contaminated plates/conditions were terminated from propagation and removed from analysis.

### Mapping of mutant strains

Mutant strains were mapped using either the VC20019 mapping strain or DM7448 (VC20019; Ex[P*myo-3*::*YFP*]). Briefly, mapping strain males were crossed with mutant hermaphrodites. 15-20 cross progeny L4 hermaphrodites were selected to a single 10 cm OP50-seeded NGM plate and grown to starvation before propagating a subpopulation to a replicate 10 cm plate. Slow growth mutants were mapped on 10 cm NGM plates seeded with OP50 and grown at 20°. ED3052 was mapped on 10cm NGM plates supplemented with 25 µg/mL carbenicillin, 4mM IPTG, and seeded with *emb-27* RNAi or HT115 bacteria. Mapping populations were propagated under selection for four to seven generations. Representative samples were chosen to extract genomic DNA as template for MIP-MAP libraries and then sequenced on Illumina MiSeq or NextSeq instruments. MIP-MAP analysis was completed as previously described (Mok *et al.* 2017). Mapping data replicates are biologically separate samples generated from F1s of the original mapping crosses.

### Head-to-head competitive fitness assessment

The head-to-head assessment of ED3052 fitness *vs.* VC20019 was conducted by first seeding 10 cm selection plates with 20 L4 animals from each strain. Populations were grown approximately 72-96 hr until brief starvation (< 24 hr) before being chunk-transferred to replicate plates for continued selection. Samples were grown in 2 selection series (*emb-27* RNAi bacteria) and 2 control series (HT115 bacteria). Plates were propagated in this manner for 11 cycles with samples collected each cycle for subsequent sequencing analysis. Samples from a subset of timepoints were made into genomic DNA and the VC20019 MIP-MAP series of probes were used to create sequencing libraries. Incompatible probes were removed from analyses before determining the mean abundances across all probes within a sample library. This value was used to represent the abundance of VC20019 within each replicate at each timepoint.

### Competitive fitness MIP library data analysis

For each specific MIP pool, reads were initially analyzed as previously described (Mok *et al.* 2017) with the exclusion of the normalization step for each MIP. After the abundance of each MIP was calculated, a mean abundance was calculated for each strain as well as a standard deviation across this mean. These values were used in downstream analysis of population structure across multiple timepoints.

Population structure and fold-change analysis was calculated across each experiment using the amalgamated data from above. Strains with a starting abundance value below 2.5x10^−3^ were eliminated from downstream population analysis. Remaining data were further transformed with any values below 1.0x10^−3^ being converted to this value to accommodate log_2_ fold-change analysis. Total fold-change and mean fold change are calculated based on starting and end-point changes in abundance *vs.* total generations (one generation per expansion). In samples with negative trajectories, however, the final generation of growth was calculated as the first instance of abundance at or below the lower limit of 1.0x10^−3^. Mean fold-change rate was calculated based on the total fold-change abundance in the final generation of growth divided by the expected number of generations passed. Each experimental condition may have had multiple biological replicates (referred to in text as replicates), each with a time-series of samples. Each fold-change rate calculated for a single time-series represented a single replicate. Mean fold-change rate for each strain was therefore a combination of multiple replicates across multiple experimental conditions (Supplemental Data SD3).

### Subgroup and principal component analysis

Comparison of intra-strain growth differences were completed by identifying pairs of conditions from pool M10 and M11 strains for which the mean fold-change rate had a difference of more than 20% between conditions. All pairs of data sets meeting the threshold difference were then statistically analyzed using Wilcoxon rank-sum test with p-values adjusted for multiple testing by the Benjamini-Hochberg method.

Principal component analysis (PCA) of pool M11 was completed by separating the data set by generation or growth condition. PCA analysis was completed in R using the prcomp() function (stats library) with data centered and scaled. Centroids represent each group as indicated by the legends, colored with information provided after PCA was completed.

### Data availability

MMP and wild isolate strains are available through the Caenorhabditis Genetics Center (CGC) and the mapping strain DM7448 is available upon request. File SD1 contains molecular inversion probe sequences and data for all 2007 MMP strains and 40 wild isolates of the Million Mutation Project. Four candidate probes for each strain were designed and listed in this file. File SD2 contains all information used in the false positive and precision analysis of PhenoMIP. File SD3 contains all mean FCR data for each strain on each replicate in each experimental pool and summaries of each strain based on pool and treatment. Custom scripts used to analyze sequencing data are available at GitHub (https://github.com/camok/PhenoMIP). Raw sequence files for pools M1 to M11 and the wild isolate pooling are available from the NIH Sequence Read Archive bioproject PRJNA595923. File SD4 contains all wild isolate pooling information including strains pooled, fold-change rates by replicate, and ED3052 mapping interval information. File SD5 contains all read depth information for each MIP within each sequencing library separated by pool and the mean abundance of each strain within each replicate separated by pool. Supplemental material available at figshare: https://doi.org/10.25387/g3.12869570.

## Results

### Molecular inversion probes reliably track multiple strains within a mixed sample

Previously, we demonstrated the usefulness of MIPs as a method to genetically map mutant alleles (Mok *et al.* 2017). In that study, our empirical analysis of MIP behavior suggested that their accuracy and precision were highest when identifying smaller subpopulations of variants. Based on this observation, we recognized that the MIP assay could be applied in a large-scale analysis of diverse compositions of strains with complex mixtures of genomic DNA. The mutagenized strains of the MMP collection presented an excellent test set. The MMP strains have an average of nearly 400 single nucleotide variants (SNVs) per strain, of which, approximately 80 are protein-coding changes (Thompson *et al.* 2013). These strains represent a unique resource for analyzing gene function on a large scale.

**Figure 1:**
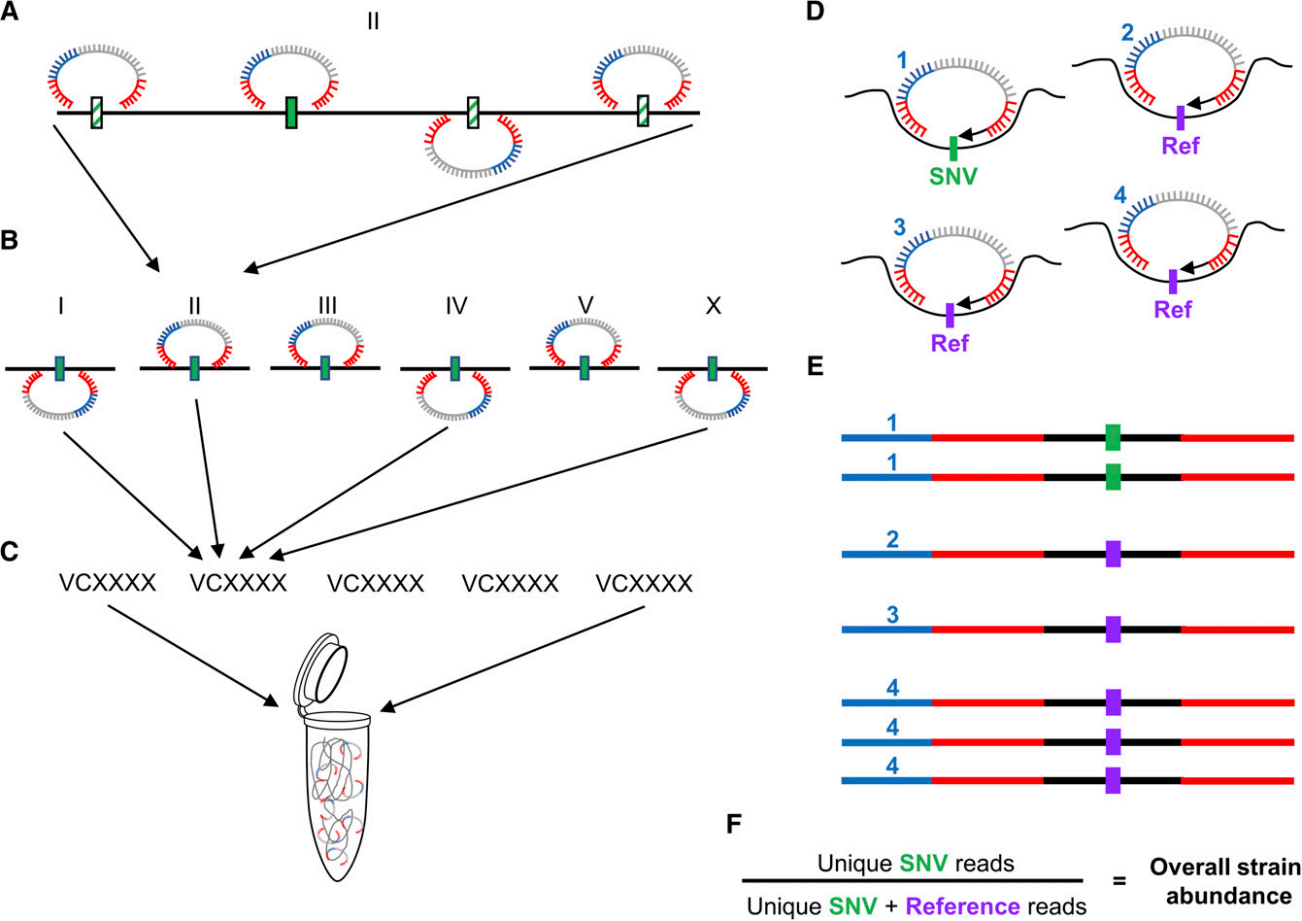
Molecular inversion probes as a system of barcoding *C. elegans* strains. MIP sequences include two annealing arms complementary to target sites (red), a unique molecular identifier (UMI, blue) and a common backbone used for library amplification and barcoding (gray). MIP sites were selected for each of 2047 MMP strains across each chromosome by excluding shared variants from all strains and then choosing sites (regardless of strain) across the genome that were separated by a minimum of 300-350bp. (A) For each strain, MIP candidate sequences were scored (solid and hatched variants). (B) The highest-scoring MIP on each chromosome (solid green) was identified. (C) Four of the six MIPs were then selected to identify a target strain among a pool of strain-specific MIPs. The MIPs would therefore have two identifiable states from the gap-fill segment of a sequencing read (D); either the strain-specific single nucleotide variant (SNV, green), or a sequence identical to the reference genome (purple). (E) After sequencing, each sample was demultiplexed by MIP target and further by the UMI to count the total number of unique annealing events specific to the SNV or reference sequences. (F) Values were compared to estimate the percentage of SNV events *vs.* the total annealing events.

As a first step we designed a set of MIPs to track strain-specific variants (Figure 1A). To avoid targeting closely spaced variants that might influence the effectiveness of individual MIP assays and to preserve the ability to make pools from any combination of MMP and wild isolate strains, we first combined variants from the 2007 mutant and 40 wild isolates strains of the entire MMP project. We eliminated shared alleles, and then chose SNVs separated by a minimum distance of 300 bp. From this list of unique candidate sites, we generated candidate MIP sequences (Mok *et al.* 2017) and for each strain we identified the highest scoring MIP sequence on each linkage group. From these top six MIPs, we assigned four representative MIPs specific to each strain (Figure 1B, and Supplemental Data SD1) with the purposes of tracking chromosomal representation in the event of cross-progeny contamination while maintaining minimal reagent costs.

To ascertain the representation of each strain in a pool, the four MIPs representing each target strain within a desired composition of strains were combined into a single pool (Figure 1C) and used in the generation of MIP sequencing libraries. The libraries were sequenced, demultiplexed and individual annealing events tracked by the unique molecular identifier (UMI) present on each oligo (Figure 1D, E). Probe sets were then combined to determine mean relative abundance for each target strain within a pooled set of genomes (Figure 1F). To successfully analyze mixed populations in an efficient high-throughput manner the PhenoMIP approach would require 1) a relatively balanced distribution of reads for each probe; 2) a low false positive rate to determine a reasonable lower bound on probe accuracy; and 3) precision between strain-specific targets to ensure that subpopulation analysis was consistent.

To test the above parameters, we generated a pool of 192 MIPs designed to target SNV sites for 48 MMP strains (Supplemental Data SD2). We generated five different sets of genomic DNA mixtures composed of subsets from 46 of the 48 MMP target strains in different proportions (two strains failed to yield adequate amounts of DNA) and used these as template samples for the generation of MIP sequencing libraries (Supplemental Data SD2). From these libraries we observed the expected composition and proportion of genotypes for the original genomic templates, suggesting that overall cross-MIP interference from multiplexing was negligible (Supplemental Figure S1A) and that the variant information from sequencing was correct. We analyzed the total number of UMIs for each MIP to gauge the efficiency of each probe. We observed eleven MIP targets that, across all libraries, consistently produced UMI counts below 20% of the mean number of UMIs per MIP in an individual library; these were removed from further analyses (Supplemental Figure S1B). To investigate the read distribution of this adjusted dataset, we normalized the UMI counts for each MIP against the minimum read number within its sequencing set. The normalized distribution of reads spanned across a ∼ninefold range with an inter-quartile range of twofold to sixfold suggesting that our distribution was relatively unimodal and ranged within a single order of magnitude (Supplemental Figure S2A and S2B).

Next, for each sequenced library, we analyzed the MIP reads from target strains that were excluded from the genomic template, calculating a total false positive rate of 1.6x10^−4^ across five MiSeq-generated data sets for which the mean UMI count per MIP was 1630 with 1.2x10^6^ unique capture events across the total set. We also compared two sequencing runs of the same PhenoMIP library with false positive rates of 1.49x10^−4^ at 3.9x10^5^ total capture events *vs.* 1.18x10^−4^ at 5.14x10^6^ total capture events. Combining all data sets we confirmed a total false positive rate of 1.25x10^−4^ across all MIPs. We estimated the mean false positive rate per individual MIP to be 1.29x10^−4^ ± 1.38x10^−4^, which compares well with our prior observations (Mok *et al.* 2017). Overall, these data suggest that the introduction of incorrect nucleotides is very infrequent across the two MIP library amplification steps of gap-filling and linearization.

When initially planning experimental design, we chose to work with pools of approximately 50 strains per set, resulting in an expected average initial population abundance of 2x10^−2^. With such a low starting abundance it was important to assess the precision between each set of strain-specific MIPs to ensure that the variation between these probes was low enough to consider their mean value a consistent assessment of strain abundance. We observed the mean standard deviation across all strain-specific MIP sets was 2.33x10^−3^ ± 6.88x10^−3^. Confirming prior observations, the absolute variance between strain-specific MIPs was dependent upon relative abundance within the sample. Subsetting the data, target strains above 5x10^−2^ abundance had a combined standard deviation between strain-specific MIPs of 1.62x10^−2^. Samples with abundance ≤ 2x10^−2^, however, had a combined standard deviation between MIPs of 2.12x10^−4^, which is similar in magnitude to our false positive rate. These findings were in line with our expectations from prior modeling of MIP behavior (Mok *et al.* 2017) (Supplemental Figure S2C).

From our analyses, we concluded that relatively consistent and balanced pools of MIPs could be generated for future analysis on complex populations; that our false positive rates remained in line with previous observations; and that overall variance among MIPs for a specific target strain was low, especially in the lower ranges of abundance. In combination with our MIP-MAP data (Mok *et al.* 2017), our analysis conservatively suggests that MIPs can accurately detect variant abundances as low as five standard deviations above the estimated false positive rate. We determined that relative abundances as low as 8.2x10^−4^ would have a high probability of being true signal as our largest false-positive value from the dataset was 7.4x10^−4^. For simplicity, we designated 1x10^−3^ as the minimum abundance required to be considered as biologically present within a given pooled population. Practically speaking, based on an average pooled experiment of 50 strains, this translates to detecting a 20-fold decrease from the expected initial abundance for a target strain. The cut-off value of 1x10^−3^ was the foundation for later analysis of our data sets with these and other MIP pools (Materials and Methods).

### MIPs identify strain fitness defects over multiple generations

**Figure 2:**
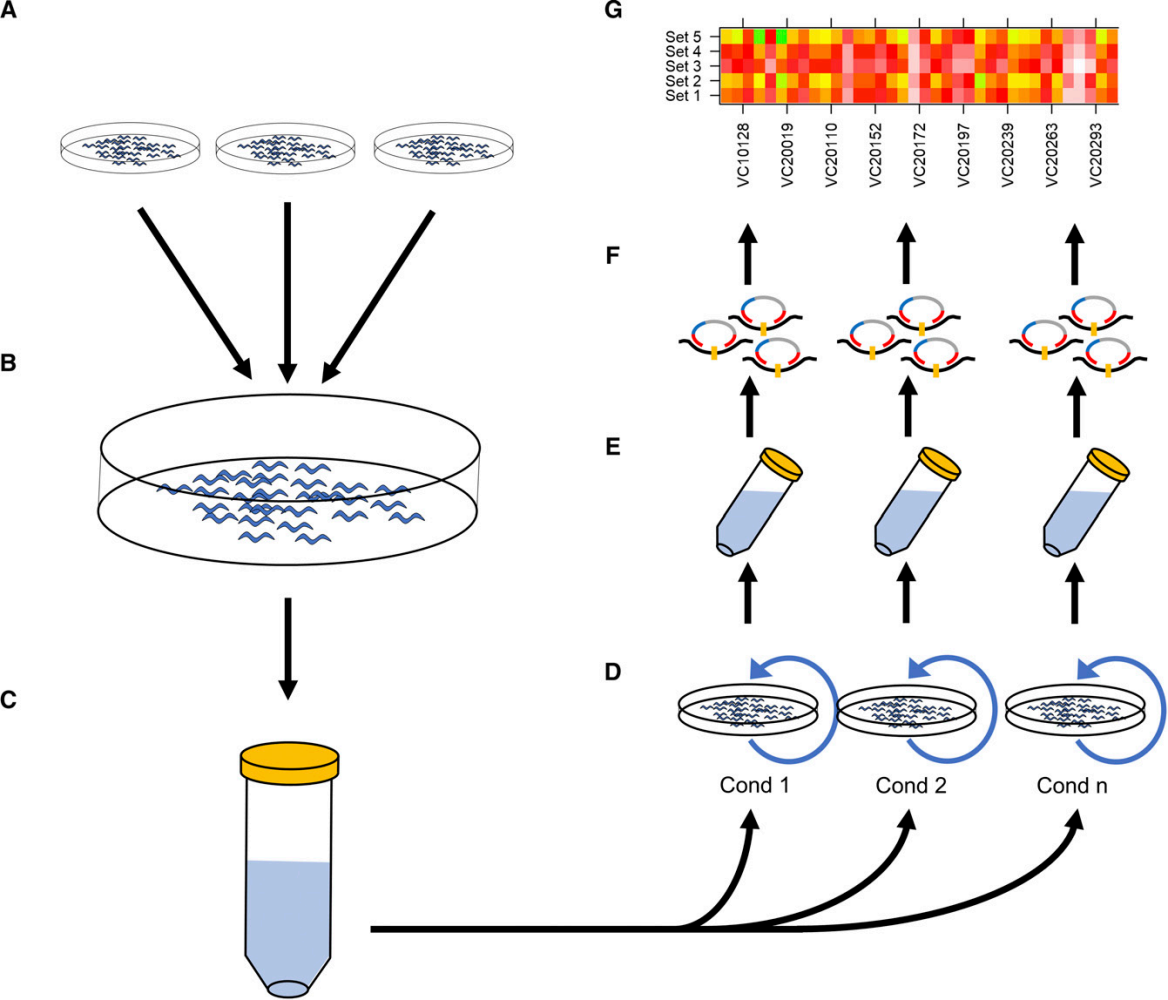
Workflow of PhenoMIP multigeneration competitive fitness assay. (A) MMP strains are selected and grown as separate populations in relative synchronization before 20 animals of each strain at the L1 (pools M1, M3, M5) or L4 stage (M7, M8, M10, M11) are transferred (B) to a communal NGM plate seeded with a bacterial lawn. The communal plates are grown in duplicate until the population has starved. (C) Uncontaminated plates are then washed and combined into a single starting population and counted for population density before being redistributed (D) onto multiple 150 mm NGM plates of varying conditions. Every 72-96 hr, the plates reach starvation and a subpopulation of animals is transferred to a new plate of the same experimental condition. (E) The remaining animals are collected for extraction of genomic DNA to generate MIP libraries for sequencing (F) and data analysis (G) of strain abundance and relative fitness.

Confident of the estimation capabilities of the MIPs, we selected sets of MMP strains to pool for growth analysis. Each pool was made up of 45-60 different MMP strains and 8-10 independent replicates were grown for multiple generations to look for differences in fitness between the strains (Table 1). In addition, to investigate the effects of different propagation methods, three food sources (*E. coli* strains HT115, NA22 or OP50) were used in different experiments and in one experiment two different methods of transfer were used (see below). The proportion of each strain in the pool was assayed at the start, terminal and various intermediate points. To ensure that a similar number of animals was present at the start and in each of the replicates (and different conditions in experiments where more than one condition was assayed), we hand-picked 20 animals from each strain at either the L1 (pool M1, M3, M5) or L4 (pool M7, M8, M10, M11) stages to duplicate *E. coli* seeded plates. Strains observed to have extremely poor population growth, or a high incidence of spontaneous males were excluded from the pooling process. We chose to grow these “starter” pools until a brief starvation of less than 24 hr (Jobson *et al.* 2015) to help hatch remaining eggs, synchronize a bulk of the population to the L1 stage and identify contamination. We then combined uncontaminated plates for an estimated 300-700K animals. This population was collected and aliquots containing 5-10K L1 animals were used to inoculate replicate cultures under their specific conditions. Cultures were grown for 72-96 hr at 20-22° (about one generation) at which point aliquots of the briefly starved populations were transferred to fresh plates. For all pools except M11, animals were transferred by chunking, while M11 replicates were split into two groups with transfer either by chunking or by washing (Table 1, Materials and Methods). This inoculation-to-starvation cycle was repeated 4-9 times, depending on the experiment. At each cycle, a fraction of the population was saved for subsequent DNA analysis (Figure 2).

**Table 1:** Summary of pooled strains

Pool name	Strains	Final sequenced generation	HT115 replicates	NA22 replicates	OP50 replicates	Combined replicates
M1	56	7	0	8	0	8
M3	57	9	0	10	0	10
M5	45	4	10	0	0	10
M7	41	4	9	0	0	9
M8	42	4	10	0	0	10
M10	60	7	10	8	6	24
M11	59	7	10 (5+5)*^a^*	8 (4+4)*^a^*	6	24
Total	**217***^b^*	**–**	**49**	**34**	**12**	**95**

a Two different methods of transfer were used for replicates.

b Total unique strains.

**Figure 3:**
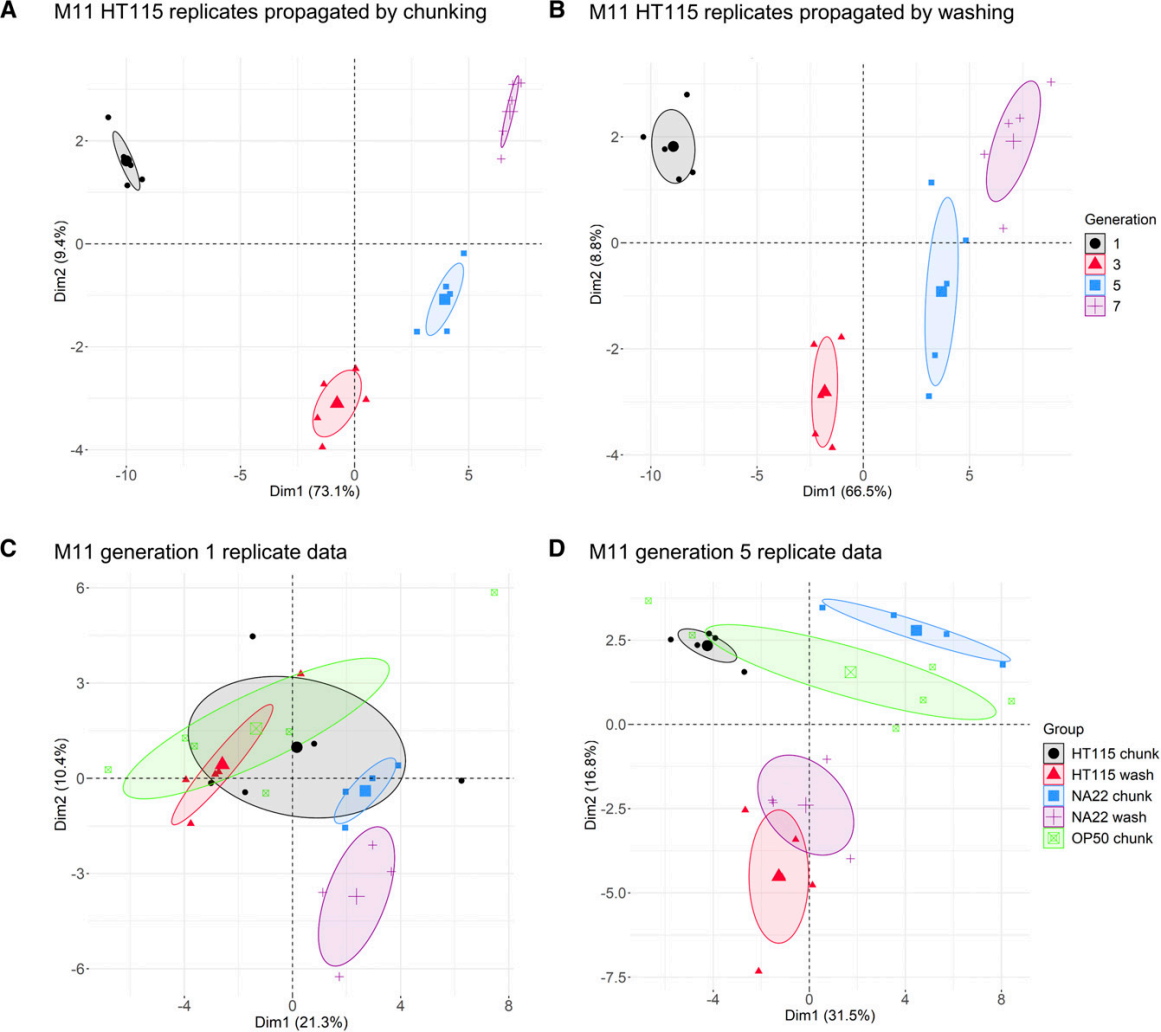
Principal component analysis of PhenoMIP data suggests consistent population stratification related to growth conditions. (A) PCA of pool M11 replicates grown on HT115 propagated by chunking and (B) M11 replicates grown on HT115 and propagated by washing are projected along principal component 1 and 2 with samples colored by generation. PCA of all pool M11 replicates from generation 1 (C) and generation 5 (D) projected along principal component 1 and 2 with samples colored by combined food source and transfer method. Large points represent the center of each cluster with the shaded area representing an ellipse with 95% confidence level.

*In toto*, we examined 217 MMP strains across seven experimental pools (Table 1, Supplemental Data SD3) to assay their relative fitness. To check the reproducibility of the data and observe overall trends we applied principal component analysis to the datasets. For example, with the M11 dataset, replicate samples with the same food source and transfer method tended to cluster tightly, but with clusters from different generations separating well after the first generation, particularly along the axis of the first principal component (Figure 3A, B and Supplemental Figure S3). Samples also separated by the methods of transfer. PCA analysis on all the M11 samples at a single timepoint shows the effect of food source as well as method of transfer over time (Figure 3C, D and Supplemental Figure S4). The OP50 replicates were not as well-correlated, and it was observed that these populations starved more quickly than other food sources. Our observations suggest that under a given experimental condition, population composition was changing with each generation in a consistent manner that was detectable by PhenoMIP analysis.

**Figure 4:**
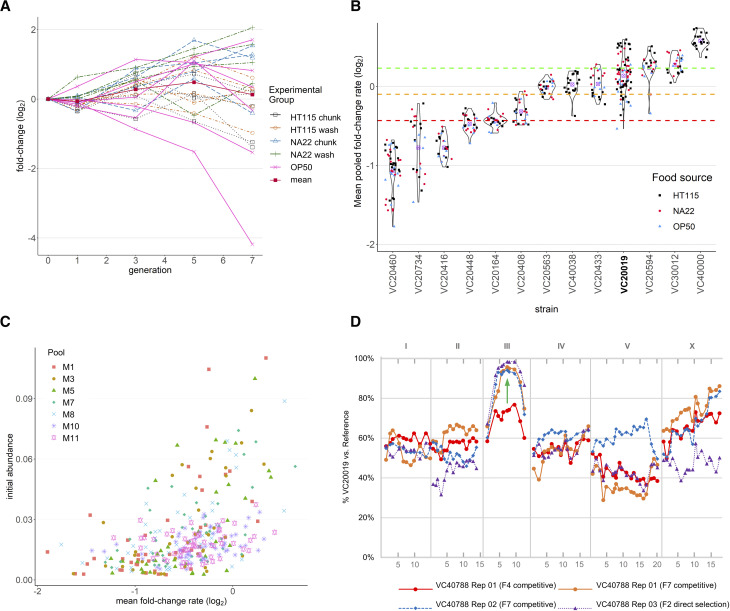
Relative fitness can be quantified by PhenoMIP and classified into subgroups. (A) Line graph of VC20019 growth rate from pool M11 with y-axis showing fold-change (log_2_) in abundance relative to initial abundance at generation 0 (starting population) across multiple generations (x-axis). Replicates are colored by experimental food source and transfer method: HT115 chunk (black squares), HT115 wash (orange circles), NA22 chunk (blue triangles), NA22 wash (green cross), OP50 chunk (pink x) and mean (mean fold change abundance across all replicates, red square). (B) Violin plots of mean fold-change per generation for a representative panel of strains. Each point represents the mean fold-change rate calculated from multiple timepoints for an experimental replicate across one or more pooling experiments. Dots are color-coded by experimental condition for growth on either HT115 (black squares), NA22 (red circles), OP50 (blue triangles) *E. coli* as a food source with overall mean fold change rate (FCR, purple ⊗). Colored dotted lines represent category boundaries using an FCR of -0.4315 (red), -0.0985 (yellow), and 0.2327 (green). VC20019 (bold) is provided as a reference for comparison to growth rates shown in (A). (C) Scatterplot of mean FCR *vs.* starting abundances (generation 0) for all strains across 7 pooled datasets. Spearman correlation across all datasets is moderate with r = 0.51, *P* < 0.0001. (D) Mapping data for VC40788, a strain observed to have poor growth rate, identified an interval of interest at III:7.6-10.8 Mbp (green arrow). Mapping was accomplished using two replicates by competitive fitness for wild type growth (orange circle and blue diamond) as well as by identifying F2 homozygous wild-type F2 recombinants in a bulk segregant assay (purple triangle). X-axis units are in megabases across each chromosome.

Confident that the assay was behaving well overall, we next assessed each strain separately for relative changes in its abundance over multiple generations across multiple replicates. For each replicate condition within a pooling experiment, this effectively created a growth profile for each strain consisting of the total fold-change and the mean fold-change rate (FCR) per generation. For example, Figure 4A plots the relative abundance of strain VC20019 in the M11 pools under various conditions. The log-fold change is modest, with the mean across nearly all conditions at close to zero, supporting that this strain is of average fitness. Closer inspection suggests that some of the variation is due to the different growth conditions used in M11, with replicates grown on NA22 and transferred by washing showing better than average growth, whereas growth on HT115 and chunk transfer grew less well. In agreement with the overall PCA analysis, growth on OP50 resulted in the most variable log-fold change. We combined results across replicates for all strains to analyze FCR as a distribution across conditions (Figure 4B and Supplemental Figure S5). We identified 15 strains that failed to thrive (class 0) in the initial pool expansion steps (initial abundance < 2.5x10^−3^) suggesting they harbored potentially strong deficits to population fitness (Supplemental Table S1). We classified the remaining 202 strains using 393 sequencing libraries across seven competitive fitness pooling experiments on 95 replicate conditions to generate profiles for 170 strains grown on the bacteria HT115, 149 strains grown on NA22, and 105 strains grown on OP50 (Supplemental Figure S6A). While we observed more subtle differences within some strains for growth on different bacteria and even for methods of transfer (Supplemental Figure S6B and C), we observed pronounced differences in growth profiles between strains and focused further analysis on this feature. We observed strains that exhibited poor growth with steep population decline suggesting fitness defects as well as strains with enhanced growth when compared to our reference strain VC20019. Based on these observations, we classified each strain into one of four classes as determined by its mean FCR across all experimental replicates (Table 2, Supplemental Data SD3). Classes were designated using a simple 10-generation growth model to calculate a final abundance (A_10_) based on the log_2_-transformed mean fold-change rate ($\bar{FCR}$) such that

**Table 2:** Mean fold-change rate summary

Class	Lower bound FCR	Upper bound FCR	Total strains	% of strains
0	NA	NA	15	6.9
1	−8.64	< -0.4315	96	44.2
2	≥ -0.4315	< -0.0985	68	31.3
3	≥ -0.0985	< 0.2327	29	13.4
4	≥ 0.2327		9	4.1

$$A_{i+1}=\;{A_{i}}^{*}2^{\bar{FCR}}$$

From our initial modeling of MIP behavior, we determined a lower limit of 1x10^−3^ on abundance within a pooled sample; we, therefore, used A_10_ cut-offs of 1x10^−3^, 1x10^−2^, 1x10^−1^ as boundaries for determining classes 1 through 4 (Supplemental Figure S5). In particular, we observed that the MMP strain VC20019, which we had previously reported as having a rate of growth similar to the lab reference strain N2 (Mok *et al.* 2017), fell into class 3 with an $\bar{FCR}$ of 0.136 or growth multiplier ($2^{\bar{FCR}}$) of 1.10 per generation (Figure 4B). Comparing the $\bar{FCR}$ across all pools against the standard deviation of the FCRs used to generate them, we observed a weak negative correlation between variation in strains with lower $\bar{FCR},$ although this effect was inconsistent between pools (Supplemental Figure S7A). In contrast, we observed strains to have a moderate positive correlation between $\bar{FCR}$ and abundance in the starting population for most pools (Figure 4C), which suggested some potential bias in the initial pooling process. Indeed, subdividing VC20019 data by experimental pool suggested there was potential for pool-specific variation (Supplement Figure S7B). The higher $\bar{FCR}$ for VC20019 in pool M8 may be a result of over-representation in the seeding population by double as this strain was also conspicuously absent from the M7 seeding population, which was pooled in parallel to M8. Our observations of the $\bar{FCR}$ across all strains suggests a wide range of fitness phenotypes across the MMP collection.

### The reduced fitness phenotypes of MMP strains were mapped to candidate mutations

Based on the results of our growth analysis, we hypothesized that underlying mutations within some strains could account for the observed growth rates. We proceeded to genetically map a subset of class 0 and class 1 strains as they exhibited the greatest reduced fitness in comparison to our control strain VC20019. We used our MIP-MAP protocol (Mok *et al.* 2017) to competitively select against the reduced fitness phenotype and identify a small genomic region containing the associated causal variant. Briefly, mutant strains were crossed with males of the mapping strain VC20019 and the population was grown until starvation. A small portion of the population was then transferred to OP50-seeded 10cm NGM plates. This transfer was completed approximately once per generation for up to 6 generations. Samples were taken at each transfer step and used to prepare genomic DNA for MIP-MAP libraries and sequencing.

We chose five class 0 and two class 1 strains to map, and successfully identified a single locus linked to a reduced population fitness for six strains (Table 3 and Supplemental Figure S8); a seventh strain appeared to have two loci. After phenotyping individual strains for possible causes of fitness defects, we were able to assign candidate alleles based on genes with shared phenotypes. In particular, we verified the mapping results of strain VC40788 by following a partially penetrant maternal-effect embryonic lethal phenotype (Figure 4D). From VC40788 and VC20019 cross progeny, we individually cultured 100 F2 animals and observed F3 and F4 progeny to specifically identify recombinant populations that failed to produce dead embryos or those that starved at the same rate as VC20019 controls. Positively identified populations were combined for MIP-MAP analysis (Materials and Methods). The primary candidate mutation for VC40788 is a G405R mutation in the mitochondrial protein B0303.3, which is predicted to have multiple functions including an acetyl-CoA C-acyltransferase activity. *B0303.3* has no reported hypomorphic or null mutant alleles but is reported to have an embryonic lethal phenotype by RNAi (Gönczy *et al.* 2000; Sönnichsen *et al.* 2005) and its human ortholog *HADHB* is implicated in trifunctional protein deficiency phenotype (Spiekerkoetter *et al.* 2003; Purevsuren *et al.* 2009). The identification of a maternal hypomorphic allele of *B0303.3* provides a means with which to study this disease and its phenotypes in a nematode model.

**Table 3:** Mapping data summary

Strain	Pools	Mean FCR	Class	Mapping Interval	Coding alleles	Likely Candidate
VC20019	All but M1	0.136	3	–	–	–
VC30079	M5, M6	−0.740	1	II:7.49-11.5 Mbp	3	*hpo-35*
				III:5.8-7.6 Mbp	3	*dig-1*
VC30188	M5, M6	−1.038	1	II:6.2-12.1 Mbp	1	*mel-11*
VC40196	M1, M3	–	0	IV:8.4-13.9 Mbp	13	*–*
VC40296	M5, M6	–	0	IV:4.2-6.4 Mbp	2	*rme-2*
VC40545	M1, M3	–	0	II:4.4-8.1 Mbp	12	*tsn-1*
VC40611	M1, M3	–	0	II:6.3-8.1 Mbp	7	*–*
VC40788	M1, M3	–	0	III:7.6-10.8 Mbp	2	*B0303.3*

### PhenoMIP of wild isolate pools can identify RNAi fitness phenotypes

**Figure 5:**
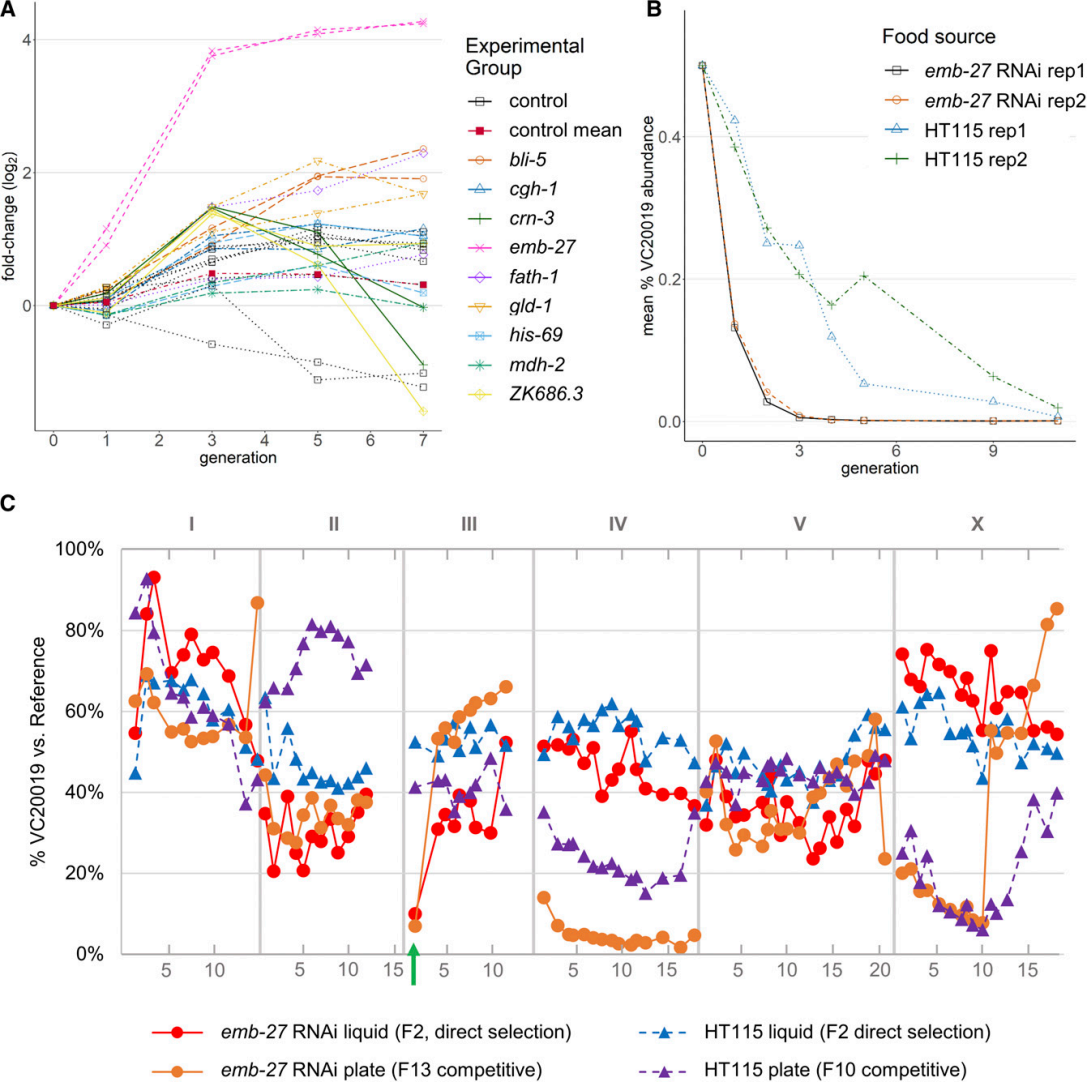
ED3052 populations exhibit suppression of *emb-27* RNAi embryonic lethal phenotype. (A) Line graph of ED3052 growth rate across multiple RNAi conditions. Growth on HT115 is represented as “control” replicates on the graph. (B) Head to head growth of ED3052 *vs.* VC20019 on *emb-27* RNAi and HT115 control plates. Y-axis represents mean abundance of VC20019 by combining MIP-MAP sequencing data across the genome into a single mean value at each timepoint. (C) MIP-MAP of ED3052 using direct F2 selection by *emb-27* RNAi phenotype (solid red circles) and using competitive fitness selection on *emb-27* RNAi over multiple time-points (solid orange circles) *vs.* their HT115-grown control counterparts (blue and purple triangles). A green arrow on LGIII indicates the loss of VC20019 genome in samples grown on *emb-27* RNAi *vs.* HT115 controls.

While the MMP mutated strains provide a diverse range of variants, the 40 wild isolates that were sequenced as part of the MMP provide a dense library of more than 600K variants from strains that have been evolving in a natural environment. We selected a subset of 27 wild isolate strains (Supplemental Data SD4) that maximized genomic diversity while reducing SNV redundancy and pooled these with VC20019. We explored the pool’s relative fitness under the selection of gene knockdown by RNAi feeding (Table 4 and Materials and Methods). Analyzing the mean FCR from our RNAi experiments, we identified a strong interaction presenting as a partial suppression of embryonic lethality phenotype in both replicate experiments of ED3052 grown on *emb-27* RNAi (Figure 5A and Supplemental Data SD4). We confirmed the observed ED3052 phenotype by growing it as a single strain on *emb-27* and in a head-to-head competition *vs.* VC20019 (Figure 5B). The *emb-27* locus of ED3052 has no coding variants and 13 variants present within the 5 kbp up- or downstream of the gene itself – all of which are shared with other wild isolate strains from the PhenoMIP analysis pool. Using the *emb-27* RNAi phenotype, we mapped this phenotype by MIP-MAP using both a competitive fitness selection and direct F2-selection protocol (Mok *et al.* 2017). Both sets of mapping data share ED3052 genomic fixation in the region from 0 Mbp to 3.6 Mbp on LG III for samples grown on *emb-27* RNAi *vs.* negative controls grown on HT115 (Figure 5C). This mapped region encompasses 158 variants unique to ED3052 among the other 26 wild isolate strains from the pooled data (Supplemental Data SD4). These changes encompass 6 protein-coding variants across 5 genes (Supplemental Table S2). In addition, there is a 66kb copy number variant deletion within this interval that encompasses 17 protein coding genes and 1 non-coding RNA. There may also be variants that were not positively identified by the MMP sequencing data due to high divergence from the N2 reference genome (Thompson *et al.* 2015). Further investigation into this interval will be required to resolve the relationship between *emb-27* RNAi suppression and ED3052. Altogether, these analyses suggest that the PhenoMIP approach can be used to dissect genomically complex pools and that PhenoMIP has enough resolution to identify intra-strain fitness changes attributed to altered growth conditions such as gene knockdown by RNAi feeding.

**Table 4:** RNAi targets for competitive growth of wild isolates

Sequence name	Gene name	Reported RNAi phenotypes
F45G2.5	*bli-5*	Adult lethal, larval lethal, reduced brood size
C07H6.5	*cgh-1*	Adult lethal, cell death variant, embryonic lethal, larval lethal, sterile
C14A4.4	*crn-3*	Embryonic lethal, larval arrest, slow growth
F10B5.6	*emb-27*	Embryonic lethal, mitosis variant, sterile
C25A1.5	*fath-1*	Larval arrest, lethal, slow growth, sterile,
T23G11.3	*gld-1*	Embryonic lethal, maternal sterile, slow growth
E03A3.3	*his-69*	embryonic lethal, organismal development variant, shortened life span, slow growth, sterile
F20H11.3	*mdh-2*	Embryonic lethal, larval arrest, reduced brood size
ZK686.3	*ZK686.3*	Embryonic lethal, larval arrest, reduced brood size, sick, sterile

^a^An additional control targeting GFP was used as a negative control in these experiments.

## Discussion

With advances in sequencing, genome-editing, and imaging, one remaining bottleneck in the characterization of the *C. elegans* genome is our ability to identify the phenotypes associated with gene function (Houle *et al.* 2010; Granier and Vile 2014). The ability to quantify population fitness along a spectrum provides a window into gene functions that may otherwise be overlooked under current experimental paradigms. Dissecting the varied contributions MMP alleles will help to generate new gene networks and build upon our understanding of worm development, reproduction, and overall fitness. With PhenoMIP, we analyzed strains from the Million Mutation Project, which offers a unique library of mutagenized genomes with coding and non-coding elements that remain largely unexplored. We efficiently identified phenotypic traits related to population fitness in a high-throughput manner by pooling multiple MMP strains in a multi-generational experiment and sequencing these populations with molecular inversion probes.

To use MIPs as a means of barcoding strains for population analysis, we designed a series of probes for the 2007 MMP strains and tested a subset on the MMP collection. We observed that we could accurately gauge a strain’s relative abundance within a sample. By sequencing multiple genomic mixtures, we confirmed a low false positive rate, suggesting we could use MIPs to accurately identify subpopulations with abundance as low as 8x10^−4^ which translates to better than 1 in 1000 genomes per sample. As a demonstration of this method, we pooled MMP strains into groups and dissected population composition over multiple generations. Our observations suggest that this form of population barcoding is indeed capable of identifying specific Million Mutation Project strains with differing levels of relative fitness. Our analysis demonstrates that PhenoMIP identifies reproducible condition-dependent population stratification among populations that have been separated for multiple generations. Based on the strains tested thus far, we estimate upwards of 82% of MMP strains may harbor alleles contributing to fitness phenotypes in the range of class 0 to class 2. Given the mutagenized and inbred nature of the MMP strains (Thompson *et al.* 2013), it is not surprising to find such an array of fitness phenotypes. These strains, however, represent a valuable resource to study fitness as the causative alleles of these effects may be in putative essential genes, poorly characterized genes with only moderate effects on fitness, or even regulatory regions of the genome. PhenoMIP has the potential to help quickly quantify these strains into relative fitness categories for researchers to prioritize their studies.

The observed population-level phenotypes presented in this work are a readout of relative fitness in a multi-strain competitive environment. Depending on selection and pooling method, weaker changes to relative fitness may be attributed to the population mixture rather than the selection variable itself (Li *et al.* 2018). Although our PhenoMIP estimates used data from multiple timepoints to estimate relative fitness within our populations, candidate strains should be validated outside the context of a pooled population study to gauge their suitability for further analysis and potential genetic or association mapping. Another factor in interpreting our data series is that pools were initially generated by combining small numbers of larval animals as a seeding parental population that was expanded before aliquoting out to replicate experiments. During the initial expansion of the seed population, the stochastic loss of even a single parental animal could impact the abundance of a strain in the initial stages of the experiment. This initial expansion is also influenced by the relative fitness of strains, which is suggested by the observation of a moderate correlation between abundance and mean fold-change rates. Similarly, we observed in our analysis of pool M8, that the doubling of VC20019 animals in the initial pooling also affected the population structure and mean fold-change rate of VC20019 itself. A potential solution to mitigate “seeding” variation would be to synchronize all of the target strains with sodium hypochlorite in the L1 larval stage (Stiernagle 2006) and then combine them in equal portions into a single population before aliquoting directly to replicate experiments. Another influence on population structure is the group of class 4 strains identified in our study. Their rapid growth and expansion can lead to drastic population stratification and the premature loss of subpopulations. In these cases, the quantitative phenotyping of less fit strains may be hindered, less informative, or potentially less accurate when analyzing a multi-generational experiment. Our observations also suggest that food source can alter population growth with food scarcity contributing to greater variation between replicates. For example, our OP50 replicates may have experienced premature starvation or uneven food distribution among populations, leading to lower population sizes and possibly affecting the consistency of the OP50-grown replicates. For an auxotrophic food source such as OP50, it would be best to concentrate cultures to generate a higher density bacterial lawn for nematode populations to consume. Lastly, the method and timing of population transfer is a potential source of selective influence. Our data suggested that chunking *vs.* washing populations to propagate them did introduce technical variation within a small subset of strains. A method of population transfer that was not addressed in this work is the sodium hypochlorite synchronization method (Stiernagle 2006), which would add the benefit of removing sporadic contamination while indirectly assaying developmental timing and fecundity. Some strains, however, may be differentially sensitive to the sodium hypochlorite solution, starvation or recovery from starvation (Baugh 2013; Webster *et al.* 2019). Over many generations, the above technical variation can amplify within the population, potentially skewing the changes observed. Therefore, when applying specific selective pressures to a population (temperature, food source, RNAi, etc.), the proper use of control conditions and replicates can help to identify the effects of technical variation with minimal impact to the sequencing burden of the experiment. Other sources of technical variation may enter during the library preparation phase via population sampling of genomic DNA, and sampling variance of the MIP library during sequencing. The use of multiple experimental replicates, sufficient sequencing depth, and careful consideration of experimental design should reduce the impact of this variation in downstream analyses.

Looking to the future, given the wide range of sequenced strains available from the Million Mutation Project and *Caenorhabditis elegans* Natural Diversity Resource (Cook *et al.* 2017), a more extensive competitive fitness assessment by PhenoMIP would set the stage for generating balanced pools of strains based on similar growth rates. That we could observe fitness differences between strains when grown on standard conditions suggests that the mutations present do exert an appreciable effect on fitness. In future experiments, balancing pooled strains by a similar predetermined fitness category would benefit long-term population analysis under varying conditions. This format would reduce fitness differences due to specific mutations and increase the likelihood of identifying condition-specific interactions such as food source or gene knockdown by RNAi-feeding. Cataloging strains of the MMP and CENDR collections will provide flexibility to future pooling strategies while identifying pre-existing fitness mutants from the collection that may also be of interest. Consequently, balanced pools could be generated randomly or based on parameters such as geographic distribution or specific genotypes or haplotypes of interest. These pools could be used to screen for phenotypic differences in conditions that alter temperature, food source (Dirksen *et al.* 2016; Zhang *et al.* 2017), resource limitation, small molecule exposure, or pathogen infection. Recently, Webster *et al.*, utilized RAD-seq techniques to assess starvation resistance on a multiplexed pool of 96 wild isolate strains (Webster *et al.* 2019). This form of competitive fitness selection is an ideal experimental context for PhenoMIP to increase potential throughput by addressing additional parameters or variables related to starvation response. Furthermore, the process of pooled competition facilitates screening on multiple strains in scenarios where the substrates or reagents tested are not easily obtained or have limited availability. In combination with GWAS and genetic mapping, PhenoMIP could prove useful in assembling a greater understanding of the many unexplored gene and regulatory sequence functions within the *C. elegans* genome.

To our knowledge these experiments are the first to use molecular inversion probes to analyze *C. elegans* populations for relative fitness. With PhenoMIP, we analyzed 217 MMP mutant strains across 95 replicate conditions and 29 timepoints for a total of 393 genomic samples. A similar analysis of our experimental data by whole genome sequencing with at least a 2000X read depth across the genome on 393 genomic samples would require between 430 and 650 300-cycle Illumina NextSeq runs. In contrast, our data were generated on the equivalent of a single 75-cycle NextSeq run. Methods such as *harp* can calculate the maximum likelihood estimates of pooled sequence data (Kessner *et al.* 2013) using haplotype blocks to reduce the amount of sequencing coverage while obtaining similar error rates to PhenoMIP, although datasets were not evaluated at abundances as low as our own datasets. Furthermore, *harp* results are most optimal when the divergence between founder genomes is 5–33% and the MMP genomes are much less divergent at an average of 400 SNVs per strain. The wild isolate strains, while more diverse, may be eligible for such methods. To match the sensitivity of PhenoMIP, samples would still require at least 200X coverage per sample or greater than 40 times the sequencing burden of PhenoMIP itself (Kessner *et al.* 2013; Tilk *et al.* 2019). As a last consideration, the design and production of MIPs as an initial sunken cost is a commitment required for investigating strains in this manner. We have included MIP sequences for the 2007 mutant and 40 wild isolate strains of the MMP (Supplemental Data SD1). Overall PhenoMIP remains a budget-conscious alternative to WGS, and its costs can be amortized if members of the community share probe and experimental development for well-characterized collections like the MMP and CeNDR. Notably, CeNDR has already designated six sets of strains that facilitate the association mapping process – which can potentially be used as experimental PhenoMIP pools to streamline the deciphering of results. PhenoMIP, however, is not without its caveats as the data generated is limited to assessing relative abundance and the variants assessed are limited to the population of strains in the experiment. We believe, however, that the initial processing steps and costs as well as the loss in sequencing complexity of genome data are outweighed by the increase in experimental throughput. Therefore, targeted sequencing by PhenoMIP complements pre-existing nematode collections and permits experimentation at a scale well beyond what is reasonably accomplished by standard WGS.

PhenoMIP has the potential to be applied beyond the MMP and wild isolate strains to the quantitative analyze of genomic variants in many contexts. Coupled with genome-editing techniques, PhenoMIP could be useful in studying allelic series or mutants of entire pathways for subtle phenotypic effects. The assay format could be converted to look at the selection of phenotypes occurring within a single event or generation, as in a bulk taxis assay or as a method for targeted genome monitoring under selective conditions. The fundamental leverage of this method is the use of MIPs to reduce the sequencing burden while maintaining informative parity with WGS formats in identifying subpopulation frequency. In doing so, we can vastly increase the throughput of population-level experimentation while minimizing costs.
